# Impact of pharmacotherapy complexity on self-care in individuals with rheumatoid arthritis

**DOI:** 10.1590/0034-7167-2024-0436

**Published:** 2025-09-08

**Authors:** Jhoniffer Lucas das Neves Matricardi, Andréia Insabralde de Queiróz-Cardoso, Carla Moreira Lorentz Higa, Guilherme Oliveira de Arruda, Maria Eduarda Gonçalves Zulin, Marcos Antonio Ferreira, Aline Fernanda Alves Ribeiro

**Affiliations:** I Universidade Federal de Mato Grosso do Sul. Campo Grande, Mato Grosso do Sul, Brazil

**Keywords:** Self Care, Drug Therapy, Arthritis, Rheumatoid, Health Promotion, Self-Management, Autocuidado, Quimioterapia, Artritis Reumatoide, Promoción de la Salud, Automanejo

## Abstract

**Objectives:**

to analyze the relationship between self-care and pharmacotherapy complexity in individuals with rheumatoid arthritis.

**Methods:**

this cross-sectional study was conducted at a teaching hospital in the Central-West region of Brazil from October to December 2023. Individuals with rheumatoid arthritis undergoing treatment for at least three months were included. A sociodemographic questionnaire, the Appraisal of Self-Care Agency Scale-Revised, and the Pharmacotherapy Complexity Index were used. Analytical statistics were applied using percentiles, the Shapiro-Wilk test, post hoc analysis, ANOVA, and the Kruskal-Wallis test.

**Results:**

the sample consisted of 96 individuals. The mean self-care and pharmacotherapy complexity scores were at intermediate levels, 56.33 and 18.29, respectively. Relationships were identified between dose frequency and self-care development, as well as between overall pharmacotherapy complexity and reduced self-care capacity.

**Conclusions:**

a high dosage frequency may positively influence self-care development in individuals with arthritis, whereas higher pharmacotherapy complexity scores may impair self-care capacity.

## RESUMEN

**Objetivos:**

analizar la relación entre autocuidado y complejidad farmacológica en indivíduos con artritis reumatoide.

**Métodos:**

estudio transversal, realizado en un hospital docente de la región Centro-Oeste, de octubre a diciembre de 2023. Se incluyeron individuos con artritis reumatoide en tratamiento durante al menos tres meses. Se utilizó formulario sociodemográfico, Escala de Evaluación de la Agencia de Autocuidado Revisada y Índice de Complejidad Farmacoterapéutica. Se utilizaron estadísticas analíticas mediante percentiles, Shapiro-Wilk, post hoc, ANOVA y Kruskal-Wallis.

**Resultados:**

la muestra estuvo constituida por 96 individuos. Medias de complejidad de autocuidado y farmacoterapia se situaron en niveles intermedios, respectivamente, 56,33 y 18,29. Se encontraron relaciones entre frecuencia de dosis y desarrollo del autocuidado, así como entre complejidad farmacológica global y falta de poder para autocuidado.

**Conclusiones:**

dosis alta puede ser positiva para desarrollo del autocuidado en individuos con artritis, mientras que puntuaciones más altas de complejidad farmacoterapéutica pueden perjudicar el poder del autocuidado.

## INTRODUCTION

Rheumatoid arthritis (RA) is a chronic inflammatory disease characterized by irreversible joint destruction in the synovial membrane following an autoimmune or infectious stimulus^(1)^. RA leads to a decline in quality of life, functional disability, and loss of productivity, factors that directly impact individuals’ self-care and may also increase healthcare system costs^(2)^.

The presence of RA requires modifications in daily activities and adaptations to both pharmacological and non-pharmacological treatments, such as diet and regular exercise, which can directly affect self-care^(3)^.

For RA treatment to be successful, it is essential to address the need for self-care and consider the impact of the disease on treatment. Therefore, knowledge of pharmacotherapy complexity, as well as the involvement of healthcare professionals and family members, are decisive factors in medication adherence and, consequently, in self-care^(4)^.

According to Dorothea Orem’s Self-Care Deficit Theory (1980), self-care is defined as the activities an individual performs to maintain, restore, or improve their health. This theory is primarily applied to chronic diseases, focusing on awareness and learning to promote a lifestyle aimed at self-development^(5)^.

Pharmacotherapy complexity is defined as the multiple characteristics of a medication regimen that can impact treatment adherence. These include the number of medications, daily doses, number of units per dose, pharmaceutical form, and additional administration instructions^(6)^.

Due to the unpredictable course of RA, as well as the use of multiple medications in therapeutic regimens for its treatment and its impact on quality of life^(7)^, further research is needed to clarify the knowledge gap regarding the relationship between self-care and pharmacotherapy complexity in individuals with RA. It is already known that people with RA require information about their treatment and medications^(4)^, but the relationship between self-care and pharmacotherapy complexity remains poorly understood. With the development of technologies to improve self-care in RA, understanding this relationship is crucial to ensuring that interventions are tailored to patients’ needs^(3)^.

## OBJECTIVES

To analyze the relationship between self-care and pharmacotherapy complexity in individuals with rheumatoid arthritis.

## METHODS

### Ethical aspects

The study was conducted in accordance with national and international ethical guidelines for research and was approved by the Research Ethics Committee of the Federal University of Mato Grosso do Sul. The approval statement is attached to this submission, in compliance with Resolution No. 466/2012 of the National Health Council, which regulates research involving human subjects in Brazil^(8)^.

The instruments used in this study, the Pharmacotherapy Complexity Index (PCI) and the Appraisal of Self-Care Agency Scale - Revised (ASAS-R), were authorized for use by their respective authors. The teaching hospital where the study was conducted provided approval and authorization for the research to be carried out directly with patients and for access to data contained in medical records.

For data collection, individuals were approached separately at the rheumatology outpatient clinic. After receiving an explanation about the study, those who agreed to participate signed the Informed Consent Form (ICF) in duplicate.

### Study design, period, and location

This was a cross-sectional, descriptive, and analytical study guided by the STROBE tool^(9)^. The study was conducted at a teaching hospital in the Central-West region of Brazil, a reference center for outpatient care of rheumatologic conditions in the state, with approximately 900 consultations per year. Data collection took place from October to December 2023 in the waiting room of the rheumatology outpatient clinic.

### Sample

Sampling was conducted by convenience. The sample size calculation was performed using G*Power 3.1.9.7 software, considering a test power of 80%, a type I error of 5%, an effect size of 0.153, and the inclusion of up to five independent variables for potential analyses adjusted by predictors, resulting in a minimum sample size of 90 individuals.

The sample was increased by 6.6% before the start of data collection to ensure an adequate number of participants, resulting in a minimum total sample of 96 individuals. Subsequently, the sample size calculation was adjusted for one-factor analysis of variance (ANOVA), considering three comparison groups (levels of pharmacotherapy complexity). The test power and the probability of type I error were maintained (80% and 5%, respectively), with the effect size value adjusted to 0.325. During the study period, approximately 150 patients with RA were seen at the outpatient clinic.

### Inclusion and Exclusion Criteria

Participants were individuals with a confirmed diagnosis of RA who were treated at the rheumatology outpatient clinic of the teaching hospital. The inclusion criteria consisted of being 18 years or older, of either sex, meeting the ICD-10 diagnostic criteria for RA, and undergoing regular treatment for at least three months at the designated outpatient clinic to ensure that initial treatment recommendations had been implemented.

The diagnosis was based on joint involvement (number of affected joints), serological tests for anti-citrullinated peptide antibody (anti-CCP) or rheumatoid factor (RF) with low or high positivity, abnormal acute-phase reactant results (erythrocyte sedimentation rate or C-reactive protein), and symptom duration exceeding six months. To confirm the diagnosis, the patient had to meet the following criteria: I) have at least one joint with clinically defined synovitis; and II) present synovitis that is not better explained by another disease^(10)^.

Individuals with severe mental disorders were excluded to prevent confounding variables^(11)^. A medical record analysis was conducted to identify the presence of mental disorders, and those diagnosed with a severe condition underwent the Mini-Mental State Examination^(12)^ as a confirmatory exclusion step. Patients who were unable to complete the questionnaire due to various clinical conditions and those with incomplete medical records regarding medication prescriptions were also excluded.

### Study Protocol

A sociodemographic and health questionnaire developed by the researchers and two validated instruments were used: the Appraisal of Self-Care Agency Scale - Revised (ASAS-R)^(13)^ and the Pharmacotherapy Complexity Index (PCI)^(14)^. To complete the PCI and validate self-reported health information, the institutional electronic medical records system was consulted.

The sociodemographic and health questionnaire included the following variables: ICD-10 classification, age, sex, marital status, religion, education level, race/ethnicity, duration of diagnosis, treatment duration, place of residence, social benefits, health insurance status, presence of chronic diseases, lifestyle habits, non-pharmacological treatment, and pain level, which was assessed using the Visual Analog Scale (VAS) for pain.

Self-care was assessed using the ASAS-R, a tool consisting of 15 questions divided into three factors: I) having the ability for self-care; II) developing the ability for self-care; and III) lacking the ability for self-care^(15)^. This instrument was translated, adapted, and validated for use in Brazil in 2014^(13)^.

Pharmacotherapy complexity was assessed using the PCI^(14)^, which is structured into three sections: A) dosage forms; B) dose frequency; and C) additional information such as administration with food, specific timing, and other instructions. The instrument was validated for use in Brazil in 2007^(16)^.

### Data Analysis and Statistics

Descriptive statistical analysis, performed using SPSS software version 20.0, was used to characterize the study sample in terms of sociodemographic, clinical, and behavioral variables, as well as to identify central and dispersion values for self-care (ASAS-R Global and by factors) and pharmacotherapy complexity (PCI Global and by factors). Absolute and relative frequencies, minimum and maximum values, means, medians, standard deviation, and percentiles were calculated. The coefficient of variation percentage was also obtained.

Percentiles were calculated stratified by age group (“under 60 years” and “60 years or older”), allowing for the categorization of pharmacotherapy complexity levels (Low, Intermediate, and High) based on global and factor-specific scores, following a methodology used in a previous study^(17)^. Values up to the 25th percentile (p25) were considered low; those above p25 and below the 75th percentile (p75) were considered intermediate; and those equal to or above p75 were considered high.

The Shapiro-Wilk test was applied to assess the normality of self-care scores, considering the observed values for each pharmacotherapy complexity category (Low, Intermediate, and High) for both PCI Global and factor-specific scores, given that the sub-samples for these categories were smaller than 50.

Based on the normality test results, mean/median comparisons were conducted using one-factor ANOVA (for normally distributed data) and the Kruskal-Wallis test (for non-normally distributed data). When differences in ASAS-R values were identified among PCI categories, post hoc analyses or multiple comparison tests (pairwise category comparisons) were performed.

Confidence intervals of 95% were calculated for estimates related to observed proportions and identified medians. A 5% significance level was adopted for mean/median comparisons.

## RESULTS

A total of 112 individuals were approached, of whom nine were not included for not meeting the diagnostic criteria for RA, and seven declined to participate in the study. The final sample consisted of 96 individuals.

The majority of participants were 60 years or older, female, of mixed race/ethnicity, had completed elementary education, were unemployed, and received social benefits. Additionally, most did not have health insurance, resided in urban areas, and lived in the state capital. Most participants reported being married or in a stable union and identified as evangelical Christians (Table 1).

**Table 1:** Sociodemographic characteristics of the sample of individuals with rheumatoid arthritis (*N* = 96), Campo Grande, Mato Grosso do Sul, Brazil, 2024

Sociodemographic Variables	n	%	95% CI
Age group			
Under 60 years	47	49.0	39.6 – 59.4
60 years or older	49	51.0	40.6 – 60.4
Sex			
Female	85	88.5	81.3 – 94.8
Male	11	11.5	5.2 – 18.8
Race/Ethnicity			
Mixed-race	60	62.5	53.1 – 70.8
White	19	19.8	12.5 – 28.1
Black	16	16.7	9.4 – 24.0
Asian	1	1.0	0.0 – 3.1
Marital status			
Single	26	27.1	18.8 – 35.4
Married/stable union	44	45.8	36.5 – 56.2
Widowed	13	13.5	7.3 – 20.8
Separated/divorced	13	13.5	7.3 – 20.8
Education level			
Illiterate	3	3.1	0.0 – 7.3
Elementary school	57	59.4	50.0 – 68.8
High school	29	30.2	20.8 – 39.6
Higher education	7	7.3	3.1 – 12.5
Religion			
None	10	10.4	5.2 – 16.7
Evangelical	43	44.8	34.4 – 55.2
Catholic	37	38.5	29.2 – 50.0
Other	6	6.3	2.1 – 11.5
Employment status			
Yes	23	24.0	16.7 – 33.3
No	73	76.0	66.7 – 83.3
Government social benefits*			
Yes	67	69.8	60.4 – 79.2
No	29	30.2	20.8 – 39.6
Health insurance			
Yes	22	22.9	14.6 – 31.3
No	74	77.1	68.8 – 85.4
Location			
Urban	92	95.8	91.7 – 99.0
Rural	4	4.2	1.0 – 8.3
City			
Capital	63	65.6	57.3 – 75.0
Countryside	33	34.4	25.0 – 42.7

*n – absolute frequency; % – relative percentage frequency; 95% CI – 95% confidence interval; government social benefits: Bolsa Família, disability assistance, or retirement.*

Among the clinical characteristics, the majority of participants met the criteria for seropositive RA (ICD-10 M05) and had at least one comorbidity (Table 2). Most participants reported experiencing frequent pain and stated that this pain interfered with their daily activities (Table 2).

**Table 2:** Clinical and behavioral characteristics of the sample of individuals with rheumatoid arthritis, Campo Grande, Mato Grosso do Sul, Brazil, 2024

Clinical and Behavioral Variables	n	%	95% CI
Type of Rheumatoid Arthritis (ICD-10)			
Seropositive (M05)	54	56.3	46.9 – 66.7
With involvement of other organs and systems (M05.3)	1	1.0	0.0 – 3.1
Other seropositive rheumatoid arthritis (M05.8)	16	16.7	9.4 – 25.0
Other rheumatoid arthritis (M06)	14	14.6	8.3 – 21.9
Seronegative (M06.0)	2	2.1	0.0 – 5.2
Other specified rheumatoid arthritis (M06.8)	2	2.1	0.0 – 5.2
Unspecified (M06.9)	7	7.3	2.1 – 12.5
Number of comorbidities			
0	10	10.4	4.2 – 16.7
1	11	11.5	5.2 – 18.8
2	18	18.8	11.5 – 27.1
3	14	14.6	7.3 – 21.9
4	21	21.9	13.5 – 30.2
5	10	10.4	4.2 – 16.7
6	3	3.1	0.0 – 7.3
7	6	6.3	2.1 – 11.5
8	2	2.1	0.0 – 5.2
10	1	1.0	0.0 – 3.1
Frequent pain			
Yes	69	71.9	61.5 – 80.2
No	27	28.1	19.8 – 38.5
Interferes with daily activities			
Yes	57	59.4	50.0 – 68.8
No	39	40.6	31.3 – 50.0
Non-pharmacological treatment			
Yes	41	42.7	33.3 – 52.1
No	55	57.3	47.9 – 66.7
Psychologist			
Yes	6	6.3	2.1 – 11.5
No	90	93.8	88.5 – 97.9
Psychiatrist			
Yes	9	9.4	4.2 – 15.6
No	87	90.6	84.4 – 95.8
Cigarette use			
Yes	17	17.7	10.4 – 25.0
No	79	82.3	75.0 – 89.6
Alcohol use			
Yes	12	12.5	6.3 – 19.8
No	84	87.5	80.2 – 93.8

*n – absolute frequency; % – relative percentage frequency; 95% CI – 95% confidence interval; ICD-10 – International Classification of Diseases, 10th revision; RA – Rheumatoid arthritis.*

Using the Visual Analog Scale (VAS) for pain assessment, it was observed that the absence of pain (0 points) was the most frequent score (n = 24; 25.0%), followed by a score of 7 (n = 14; 14.6%) and a score of 8 (n = 12; 12.5%). The mean score was 4.63 (standard deviation = 3.41; median = 5.0; minimum = 0.0; maximum = 10) (results not tabulated).

The majority of participants did not undergo non-pharmacological treatments nor had psychological or psychiatric follow-up. In terms of behavioral characteristics, most participants did not use tobacco or alcohol.

Table 3 presents a description of central tendency and dispersion measures for global and factor-specific values of self-care and pharmacotherapy complexity.

**Table 3:** Description of self-care and pharmacotherapy complexity scores for the sample of individuals with rheumatoid arthritis, Campo Grande, Mato Grosso do Sul, Brazil, 2024

Variable	Mean	Med.	SD	CV%	Min	Max	P25	p75
ASAS-R Global	56.33	57.0	8.62	15.30	36	75	50.25	62.0
ASAS-R – Factor 1	24.31	24.0	4.44	18.26	8	30	22.0	28.0
ASAS-R – Factor 2	20.54	21.0	3.30	16.06	10	25	18.25	23.0
ASAS-R – Factor 3	11.48	11.0	3.57	31.09	4	20	9.0	14.0
ICFT Global	18.29	16.0	8.75	47.84	2	44	12.0	23.0
ICFT – Factor 1	3.05	3.0	2.28	74.75	1	11	1.0	5.0
ICFT – Factor 2	12.34	12.0	5.98	48.46	1	32	8.0	16.0
ICFT – Factor 3	2.96	2.0	2.98	100.6	0	12	1.0	4.0

*ASAS-R – Appraisal of Self-Care Agency Scale – Revised; ICFT – Pharmacotherapy Complexity Index; Med – Median; SD – Standard Deviation; CV – Coefficient of Variation Percentage; p25 – 25th Percentile (1st quartile); p75 – 75th Percentile (3rd quartile).*

The p25 and p75 values for adults were as follows: 11.0 and 22.0 for the Global score; 1.0 and 5.0 for Factor 1; 7.0 and 14.0 for Factor 2; and 0.0 and 4.0 for Factor 3. For older adults, the identified values were: 13.0 and 25.5 for the Global score; 1.0 and 5.0 for Factor 1; 8.5 and 18.0 for Factor 2; and 1.0 and 4.0 for Factor 3.

Overall, most of the sample exhibited intermediate pharmacotherapy complexity. The same trend was observed when analyzing Factor 2 scores of the ICFT separately. For Factor 1, most of the sample fell within the low complexity category, whereas for Factor 3, most participants ranged between intermediate and high complexity (Table 4).

**Table 4:** Frequencies of pharmacotherapy complexity levels according to ICFT Global and factors for the sample of individuals with rheumatoid arthritis, Campo Grande, Mato Grosso do Sul, Brazil, 2024

ICFT	Low Complexity			Intermediate Complexity			High Complexity		
	n	%	95% CI	n	%	95% CI	n	%	95% CI
Global	29	30.2	21.9 – 39.6	46	47.9	37.5 – 58.3	21	21.9	13.5 – 31.3
Factor 1	46	47.9	38.5 – 58.3	22	22.9	14.6 – 31.3	28	29.2	19.8 – 37.5
Factor 2	26	27.1	18.8 – 36.5	44	45.8	35.4 – 55.2	26	27.1	17.7 – 35.4
Factor 3	30	31.3	21.9 – 41.6	33	34.4	25.0 – 43.8	33	34.4	25.0 – 43.8

*ICFT – Pharmacotherapy Complexity Index; n – absolute frequency; % – relative percentage frequency; 95% CI – 95% confidence interval.*

As shown in Table 5, significant differences were observed in the median self-care scores for Factors 2 and 3 of the ASAS-R. These differences were specifically found between individuals with Low and High levels in Factor 2 of the ICFT (p = 0.042) and between Intermediate and High levels for the ICFT Global score (p = 0.011).

**Table 5:** Relationship between self-care and pharmacotherapy complexity for the sample of individuals with rheumatoid arthritis, Campo Grande, Mato Grosso do Sul, Brazil, 2024

ICFT Variables	Self-Care Score (ASAS-R) by Levels of Pharmacological Complexity						*p* value
	Low		Intermediate		High		
	Mean (SD)	Med.	Mean (SD)	Med.	Mean (SD)	Med.	
	**ASAS-R Global**						
Global	55.62 (7.88)	57.00	57.09 (9.19)	59.50	55.67 (8.56)	55.00	0.718*
Factor 1	55.78 (9.12)	57.50	55.91 (7.93)	57.00	57.57 (8.47)	58.00	0.669*
Factor 2	55.12 (7.65)	55.00	56.68 (8.83)	57.50	56.96 (9.36)	57.50	0.699*
Factor 3	57.73 (7.99)	59.50	56.64 (9.01)	58.00	54.76 (8.78)	54.00	0.384*
	**ASAS-R – Factor 1**						
Global	24.52 (3.81)	24.00	23.91 (5.13)	24.00	24.90 (3.67)	25.00	0.891**
Factor 1	23.67 (5.19)	24.00	25.05 (3.37)	25.00	24.79 (3.77)	25.50	0.713**
Factor 2	24.27 (3.96)	24.00	24.14 (4.47)	24.00	24.65 (4.97)	26.00	0.663**
Factor 3	25.47 (3.68)	26.50	24.33 (4.53)	25.00	23.24 (4.82)	24.00	0.167**
	**ASAS-R – Factor 2**						
Global	19.69 (3.54)	20.00	20.96 (2.78)	20.50	20.81 (3.92)	21.00	0.247**
Factor 1	20.26 (3.29)	20.00	20.41 (3.34)	21.00	21.11 (3.34)	22.00	0.462**
Factor 2	19.73 (2.76)	19.50	20.41 (3.49)	20.50	21.58 (3.32)	21.50	0.048**
Factor 3	20.17 (3.45)	20.00	20.82 (3.16)	21.00	20.61 (3.37)	21.00	0.731**
	**ASAS-R – Factor 3**						
Global	11.41 (2.59)	11.00	12.22 (3.88)	12.00	9.95 (3.68)	9.00	0.015**
Factor 1	11.85 (3.26)	12.00	10.45 (3.55)	10.00	11.68 (4.02)	11.00	0.307*
Factor 2	11.12 (3.05)	10.00	12.14 (3.72)	12.00	10.73 (3.73)	10.50	0.237*
Factor 3	12.10 (3.43)	12.00	11.48 (3.65)	11.00	10.91 (3.63)	10.00	0.422*

ICFT – Pharmacotherapy Complexity Index; ASAS-R – Appraisal of Self-Care Agency Scale – Revised; *One-factor ANOVA; *Kruskal-Wallis.

Analyzing the 95% CI values for the medians in each comparison where a significant difference was identified, it is observed that the intervals overlap in the case of results related to Factor 2 of the ASAS-R and Factor 2 of the ICFT (Low = 18.00 - 21.00 vs. High = 20.51 – 24.00). This suggests that individuals with a higher dose frequency may have a greater ability to develop self-care.

On the other hand, regarding the relationship between Factor 3 of the ASAS-R and the ICFT Global score, this overlap does not occur (Intermediate = 11.00 – 14.00 vs. High = 8.00 – 10.00), reinforcing the existence of a significant difference between these groups. Thus, high overall pharmacotherapy complexity may contribute to a greater self-care deficit.

## DISCUSSION

Some sociodemographic characteristics present in the study sample include advanced age, female sex, elementary education level, presence of comorbidities, and difficulties in pain management. It is worth noting that these characteristics have also been reported in a previous study conducted in Portugal and may be associated with pharmacotherapy complexity, which can impact self-care due to impairments in functional autonomy^(18)^.

The majority of individuals were 60 years or older, undergoing polypharmacy, and had multiple comorbidities. Evidence suggests that these factors are associated with an increased likelihood of medication therapy-related problems^(19)^, as the use of multiple medications may be linked to non-adherence behaviors^(20)^, in addition to potentially influencing self-care.

It is important to highlight that the presence of comorbidities such as hypertension and diabetes (with incidences of 50% and 27%, respectively, in the sample) can increase pharmacotherapy complexity due to the introduction of different pharmaceutical forms, dosing frequencies, and additional instructions^(21)^. Besides high complexity, other challenges can compromise pharmacotherapeutic treatment and are not covered by the ICFT, such as confusion between medications, the need for reminders, and dependence on a caregiver or family assistance^(22)^.

The relationship between the ASAS-R Global score and the ICFT Global score was not statistically significant. However, a significant association was identified between Factor 2 (dose frequency) of the ICFT and Factor 2 (developing the ability for self-care) of the ASAS-R (*p* = 0.048). The difference was observed between the Low and High levels in Factor 2 (dose frequency) of the ICFT and Factor 2 of the ASAS-R (developing the ability for self-care) (*p* = 0.042). Thus, it is suggested that a high dose frequency is positively related to self-care in individuals with RA.

Evidence indicates that individuals with chronic conditions and other comorbidities report daily difficulties due to the need for frequent medication doses^(23)^. Although often perceived negatively, a treatment regimen with a high dose frequency may, in practice, promote self-care.

A significant association was also observed between the ICFT Global score and Factor 3 (lacking the ability for self-care) of the ASAS-R (*p* = 0.015). Another significant relationship was identified between Factor 3 of the ASAS-R (lacking the ability for self-care) and the intermediate and high levels of the ICFT Global score (*p* = 0.011). This suggests that a high number of medications, higher dose frequencies, and a greater amount of additional information may contribute to a deficit in self-care ability.

The literature suggests that high complexity in therapeutic regimens may hinder the adoption of self-care behaviors^(24)^. However, among home-dwelling elderly individuals, a higher level of pharmacotherapy complexity may be beneficial in fostering self-care and medication adherence, owing to the support provided by family members in maintaining drug treatment^(25)^.

A high level of pharmacotherapy complexity has been associated with lower medication adherence^(26)^. The literature highlights that greater adherence to medication can be a positive influencing factor in self-care^(27)^, making adherence a potentially important element in analyzing this relationship^(28)^.

Modifying the factors contributing to pharmacotherapy complexity can be challenging and should involve interaction with a multidisciplinary team, which plays a fundamental role in the decision-making process regarding medications^(29)^. The use of simplification strategies to reduce pill burden and dosing frequency has been reported and has shown positive results, indicating that adherence and clinical outcomes improve after regimen simplification, particularly with injectable medications^(30)^. Additionally, automating the ICFT through software can optimize its use in multidisciplinary care routines, allowing any professional to use it and obtain results quickly, facilitating early guidance for pharmacotherapy simplification in individuals with greater complexity^(31)^.

The values obtained through the ASAS-R indicate an intermediate level of self-care, similar to that identified in another study conducted with patients with fibromyalgia (another rheumatologic condition)^(32)^. The likely obstacle preventing this score from reaching a high level is the impact of the disease, which primarily manifests as pain, fatigue, and joint deformities^(33)^. These factors impair quality of life, which, in individuals with RA, is lower than that of the general population^(34)^.

To enhance self-care, it is essential to understand that motivation is linked to self-management skills. An individual with positive beliefs in their own abilities can manage specific problems. The pursuit of symptom relief and the return to daily activities are important factors that drive self-care^(35)^.

Future research on individuals with RA should aim to develop interventions focused on improving the self-care factors assessed by the ASAS-R and pharmacotherapy complexity, as well as evaluating the application of therapeutic regimen simplification strategies.

### Study limitations

This study has some limitations, as the collected data may not exhibit a high level of randomness. Expanding the sample size may have mitigated this limitation to some extent. The data do not allow for the determination of a direct relationship between the ASAS-R Global score and the ICFT Global score; however, factor-based analysis identified points of association between the scales. Additionally, there are a limited number of studies exploring this relationship, which prevented comparisons with other findings. For some comparisons, it was necessary to refer to studies on conditions similar to RA, such as fibromyalgia.

### Contributions to Nursing, Health, or Public Policy

In nursing practice, with a focus on self-care, individuals with a high dose frequency exhibit greater vulnerability in developing self-care, while those with higher levels of pharmacotherapy complexity may experience a deficit in self-care capacity.

When identifying elderly individuals with multiple comorbidities, polypharmacy, and high pharmacotherapy complexity, nurses should seek alternatives to modify this level of complexity. Implementing interventions with the multidisciplinary team and utilizing simplification strategies can be valuable tools in nursing practice.

In education, integrating the analysis of pharmacotherapy complexity and the use of interventions for its reduction, both theoretically and practically, can prepare students to address and resolve cases involving high pharmacotherapy complexity.

## CONCLUSIONS

Individuals with higher pharmacotherapy complexity scores (number of medications, dose frequency, and additional instructions) may experience a decline in self-care capacity. Conversely, a higher dose frequency, without elevated scores for the number of medications and additional instructions, may promote self-care development in individuals with RA.

Polypharmacy, comorbidities, and other demographic and clinical variables may be negatively associated with self-care and pharmacotherapy complexity. Interventions with the multidisciplinary team and the use of therapeutic regimen simplification strategies may be effective alternatives to reduce the ICFT and improve self-care in patients with RA.
