# Supplementary material for: Impact of pharmacotherapy complexity on self-care in individuals with rheumatoid arthritis
Source: Rev Bras Enferm. 2025 Sep 8;78(3):e20240436. doi: 10.1590/0034-7167-2024-0436 (PMC12419784; doi:10.1590/0034-7167-2024-0436)
Supplement: Translation

# Translation: Impacto da complexidade da farmacoterapia no autocuidado de indivíduos com artrite reumatoide

**DOI:** 10.1590/0034-7167-2024-0436pt

**Authors:** Jhoniffer Lucas das Neves Matricardi, Andréia Insabralde de Queiróz-Cardoso, Carla Moreira Lorentz Higa, Guilherme Oliveira de Arruda, Maria Eduarda Gonçalves Zulin, Marcos Antonio Ferreira, Aline Fernanda Alves Ribeiro

**Affiliations:** I Universidade Federal de Mato Grosso do Sul. Campo Grande, Mato Grosso do Sul, Brasil

**Keywords:** Autocuidado, Tratamento Farmacológico, Artrite Reumatoide, Promoção da Saúde, Autogestão

## Abstract

**Objetivos:**

analisar a relação entre autocuidado e a complexidade farmacológica em indivíduos com artrite reumatoide.

**Métodos:**

estudo transversal, realizado em um hospital-escola da região Centro-Oeste, de outubro a dezembro de 2023. Foram incluídos indivíduos com artrite reumatoide em tratamento há pelo menos três meses. Utilizou-se um formulário sociodemográfico, o *Appraisal of Self-Care Agency Scale-Revised* e o Índice de Complexidade da Farmacoterapia. Foi empregada estatística analítica por percentis, Shapiro-Wilk, post hoc, ANOVA e Kruskal-Wallis.

**Resultados:**

a amostra foi composta por 96 indivíduos. As médias para autocuidado e complexidade da farmacoterapia estavam em níveis intermediários, respectivamente, 56,33 e 18,29. Foram encontradas relações entre frequência das doses e desenvolvimento do autocuidado, bem como entre complexidade farmacológica global e falta de poder para o autocuidado.

**Conclusões:**

uma alta dosagem pode ser positiva para desenvolvimento do autocuidado em indivíduos com artrite, enquanto escores mais elevados de complexidade da farmacoterapia podem prejudicar o poder para autocuidado.

## INTRODUÇÃO

A artrite reumatoide (AR) é uma doença inflamatória crônica caracterizada pela destruição articular irreversível na membrana sinovial após um estímulo de origem autoimune ou infecciosa^(1)^. A AR leva à piora da qualidade de vida, incapacidade funcional e perda de produtividade, fatores que impactam diretamente o autocuidado dos indivíduos e também podem gerar aumento dos gastos para o sistema de saúde^(2)^.

A presença da AR exige modificações nas atividades de vida diária e adaptações ao tratamento medicamentoso e não medicamentoso, como dieta e prática regular de exercícios, que podem impactar diretamente o autocuidado^(3)^.

Para que o tratamento da AR seja bem-sucedido, é necessário abordar a necessidade de autocuidado e considerar o impacto da doença no tratamento. Por isso, o conhecimento sobre a complexidade da farmacoterapia, bem como a participação dos profissionais e da família, são fatores decisivos na adesão medicamentosa e, por consequência, no autocuidado^(4)^.

O autocuidado, segundo a Teoria do Déficit de Autocuidado de Dorothea Orem (1980), é definido como as atividades que o indivíduo realiza para manter, restaurar ou melhorar sua saúde. Essa teoria tem seu uso aplicado principalmente em doenças crônicas, com enfoque na conscientização e no aprendizado para a promoção de um estilo de vida voltado ao autodesenvolvimento^(5)^.

A complexidade da farmacoterapia é definida como as múltiplas características do tratamento medicamentoso, que podem impactar a adesão ao tratamento. São elas: número de medicamentos, quantidade de doses diárias, número de unidades por dose, forma farmacêutica e instruções adicionais de administração^(6)^.

Devido ao curso incerto da AR, assim como à utilização de múltiplos medicamentos no regime terapêutico para seu tratamento e ao impacto na qualidade de vida^(7)^, faz-se necessário o desenvolvimento de pesquisas para elucidar a lacuna do conhecimento na relação entre a condição de autocuidado de indivíduos com AR e a complexidade farmacológica. Já se sabe que pessoas com AR têm necessidade de informações sobre o tratamento e os medicamentos^(4)^, mas a relação entre o autocuidado e a complexidade farmacológica ainda é pouco elucidada. Com o desenvolvimento de tecnologias para a melhora do autocuidado na AR, a compreensão dessa relação é vital para que essas intervenções sejam informadas pelas necessidades dos pacientes^(3)^.

## OBJETIVOS

Analisar a relação entre o autocuidado e a complexidade farmacológica em indivíduos com artrite reumatoide.

## MÉTODOS

### Aspectos éticos

O estudo foi conduzido de acordo com as diretrizes éticas nacionais e internacionais para pesquisas, tendo sido aprovado pelo Comitê de Ética em Pesquisa da Universidade Federal de Mato Grosso do Sul, cujo parecer está anexado à presente submissão, em conformidade com a Resolução nº 466/2012 do Conselho Nacional de Saúde, que regulamenta a pesquisa com seres humanos no Brasil^(8)^.

Os instrumentos utilizados nesta pesquisa, o Índice de Complexidade da Farmacoterapia (ICFT) e o *Appraisal of Self-Care Agency Scale-Revised* (ASAS-R), foram liberados para uso por seus respectivos autores. O hospital-escola, local do estudo, forneceu aprovação e autorização para a execução da pesquisa diretamente com os pacientes e para a consulta aos dados contidos nos prontuários.

Para a coleta de dados, os indivíduos foram abordados de forma individualizada no ambulatório de reumatologia e, após explicações sobre a pesquisa, aqueles que concordaram em participar assinaram o Termo de Consentimento Livre e Esclarecido em duas vias.

### Desenho, período e local do estudo

Trata-se de um estudo transversal, descritivo e analítico, norteado pela ferramenta STROBE^(9)^. O estudo foi realizado em um hospital-escola da região Centro-Oeste do Brasil, referência no atendimento ambulatorial de condições reumatológicas no estado, com cerca de 900 atendimentos por ano. A coleta de dados ocorreu de outubro a dezembro de 2023 na sala de espera do ambulatório de reumatologia.

### Amostra

A amostragem ocorreu por conveniência. O cálculo amostral foi realizado por meio do software G*Power 3.1.9.7, considerando um poder do teste de 80%, erro do tipo I de 5%, *effect size* de 0,153 e inclusão de até cinco variáveis independentes para eventuais análises ajustadas pelos preditores, resultando em uma amostra mínima de 90 indivíduos.

A amostra foi ampliada em 6,6% antes do início da coleta de dados para garantir um número adequado de participantes, totalizando uma amostra mínima de 96 indivíduos. Posteriormente, ajustou-se o cálculo amostral para a análise de variância (ANOVA) de um fator, considerando três grupos de comparação (níveis de complexidade da farmacoterapia). O poder do teste e a probabilidade de erro do tipo I foram mantidos (80% e 5%, respectivamente), com ajuste do valor de *effect size* para 0,325. Durante o período do estudo, aproximadamente 150 pacientes com AR foram atendidos no ambulatório.

### Critérios de inclusão e exclusão

Os participantes foram indivíduos com diagnóstico confirmado de AR atendidos no ambulatório de reumatologia do hospital-escola. Foram considerados critérios de inclusão: ter idade igual ou superior a 18 anos, de ambos os sexos, com critérios de diagnóstico do CID-10 para AR e tratamento regular há pelo menos três meses no referido ambulatório, de modo a garantir que o início das recomendações de tratamento tenha sido realizado.

O diagnóstico tem como base o envolvimento articular (número de articulações acometidas), as provas sorológicas de anticorpo contra peptídeo citrulinado (anti-CCP) ou fator reumatoide (FR) baixo positivo ou alto positivo, resultados anormais dos reagentes de fase aguda (velocidade de hemossedimentação ou proteína C reativa) e a duração dos sinais/sintomas superior a seis meses. Para confirmação, o paciente deve: I) ter pelo menos uma articulação com sinovite clínica definida; e II) apresentar sinovite não explicada por outra doença^(10)^.

Foram excluídos indivíduos com transtornos mentais severos, para evitar influências das variáveis^(11)^. Foi realizada ainda a análise de prontuário para identificar a presença de transtornos mentais, e aqueles que apresentavam algum transtorno severo passaram pela aplicação do Mini-Exame do Estado Mental^(12)^ como etapa confirmatória para exclusão. Também foram excluídos os pacientes incapazes de responder ao formulário em decorrência de condições clínicas diversas e os prontuários incompletos em relação à prescrição médica.

### Protocolo do estudo

Foram utilizados um formulário sociodemográfico e de saúde desenvolvido pelos pesquisadores e dois instrumentos validados: o ASAS-R^(13)^ e o ICFT^(14)^. Para o preenchimento do ICFT e a validação das informações de saúde relatadas, houve consulta ao sistema de prontuários eletrônicos institucionais.

O formulário sociodemográfico e de saúde foi composto pelas seguintes variáveis: CID-10, idade, sexo, estado civil, religião, grau de instrução, cor/raça, tempo de diagnóstico, tempo de tratamento, local de residência, benefícios sociais, plano de saúde, presença de doenças crônicas, hábitos de vida, tratamento não farmacológico e nível de dor, avaliado pela Escala Visual Analógica (EVA) da dor.

O autocuidado foi avaliado por meio do ASAS-R, que é uma ferramenta composta por 15 questões, divididas em três fatores: I) tendo poder para o autocuidado; II) desenvolvendo poder para o autocuidado; e III) faltando poder para o autocuidado^(15)^. O instrumento foi traduzido, adaptado e validado em 2014 para uso no Brasil^(13)^.

A complexidade da farmacoterapia foi avaliada por meio do ICFT^(14)^, estruturado em três seções: A) sobre as formas de dosagem; B) sobre a frequência das doses; e C) informações adicionais, como uso com alimentos, horário específico e outros. O instrumento foi validado para a realidade brasileira em 2007^(16)^.

### Análise dos resultados e estatísticas

A análise estatística descritiva, realizada por meio do programa SPSS versão 20.0, foi empregada para caracterizar a amostra de participantes do estudo, considerando variáveis sociodemográficas, clínicas e comportamentais, bem como identificar valores centrais e de dispersão para o autocuidado (*ASAS-R Global* e por fatores) e para a complexidade da farmacoterapia (*ICFT Global* e por fatores). Para tanto, foram utilizadas frequências absolutas e relativas, valores mínimo e máximo, médias, medianas, desvio padrão e percentis. Também foram obtidos os valores do coeficiente de variação percentual.

Os percentis foram calculados de forma estratificada por faixa etária (“menos de 60 anos” e “60 anos ou mais”), permitindo a categorização em níveis de complexidade farmacológica (Baixa, Intermediária e Alta) a partir das pontuações globais e por fatores, conforme metodologia utilizada em outro estudo^(17)^. Os valores posicionados até o percentil 25 (p25) foram considerados baixos; aqueles superiores ao p25 e inferiores ao percentil 75 (p75), intermediários; e os iguais ou superiores ao p75, altos.

Aplicou-se o teste de Shapiro-Wilk para avaliar a normalidade das pontuações do autocuidado, considerando os valores observados para cada categoria de nível de complexidade da farmacoterapia (Baixa, Intermediária e Alta), tanto para o *ICFT Global* quanto por fatores, visto que as subamostras dessas categorias eram menores que 50.

Com base nos resultados do teste de normalidade, definiu-se a análise de comparação de médias/medianas por meio da ANOVA de um fator (para dados com distribuição normal) e do teste de Kruskal-Wallis (para dados sem distribuição normal). Quando foram identificadas diferenças nos valores do *ASAS-R* para o conjunto das categorias de *ICFT,* foram realizadas análises *post hoc* ou testes de comparações múltiplas (par a par entre as categorias).

Foram calculados intervalos de confiança de 95% para as estimativas referentes às proporções observadas e às medianas identificadas. O nível de significância adotado para as comparações de médias/medianas foi de 5%.

## RESULTADOS

Foram abordados 112 indivíduos, dos quais nove não foram incluídos por não atenderem aos critérios diagnósticos para AR, e sete recusaram participar do estudo. A amostra final foi composta por 96 indivíduos.

A maioria dos participantes tinha 60 anos ou mais, era do sexo feminino, de cor/raça parda, com ensino fundamental completo, não trabalhava e recebia benefícios sociais. Além disso, a maioria não possuía plano de saúde, residia em área urbana e na capital do estado. A maior parte dos participantes relatou ser casada ou estar em união estável e se declarar adepta da religião evangélica (Tabela 1).

**Tabela 1:** Caracterização sociodemográfica da amostra de pessoas com artrite reumatoide (*N* = 96), Campo Grande, Mato Grosso do Sul, Brasil, 2024

Variáveis sociodemográficas	n	%	IC95%
Faixa etária			
Menos de 60 anos	47	49,0	39,6 – 59,4
60 anos ou mais	49	51,0	40,6 – 60,4
Sexo			
Feminino	85	88,5	81,3 – 94,8
Masculino	11	11,5	5,2 – 18,8
Cor/raça			
Parda	60	62,5	53,1 – 70,8
Branca	19	19,8	12,5 – 28,1
Negra	16	16,7	9,4 – 24,0
Amarela	1	1,0	0,0 – 3,1
Estado civil			
Solteiro	26	27,1	18,8 – 35,4
Casado/união estável	44	45,8	36,5 – 56,2
Viúvo	13	13,5	7,3 – 20,8
Separado/divorciado	13	13,5	7,3 – 20,8
Grau de instrução			
Analfabeto	3	3,1	0,0 – 7,3
Fundamental	57	59,4	50,0 – 68,8
Médio	29	30,2	20,8 – 39,6
Superior	7	7,3	3,1 – 12,5
Religião			
Não	10	10,4	5,2 – 16,7
Evangélica	43	44,8	34,4 – 55,2
Católica	37	38,5	29,2 – 50,0
Outros	6	6,3	2,1 – 11,5
Trabalha			
Sim	23	24,0	16,7 – 33,3
Não	73	76,0	66,7 – 83,3
Benefícios sociais do governo*			
Sim	67	69,8	60,4 – 79,2
Não	29	30,2	20,8 – 39,6
Plano de saúde			
Sim	22	22,9	14,6 – 31,3
Não	74	77,1	68,8 – 85,4
Localidade			
Urbana	92	95,8	91,7 – 99,0
Rural	4	4,2	1,0 – 8,3
Cidade			
Capital	63	65,6	57,3 – 75,0
Interior	33	34,4	25,0 – 42,7

n – frequência absoluta; % – frequência relativa percentual; IC95% – intervalo de confiança de 95%; *benefícios sociais do governo: Bolsa Família, auxílio-doença ou aposentadoria.

Dentre as características clínicas, verificou-se que a maioria dos participantes se enquadrava no diagnóstico de AR soropositiva (CID-10 M05) e possuía alguma comorbidade (Tabela 2). A maior parte dos participantes relatou sentir dor com frequência e afirmou que essa dor interferia nas atividades diárias (Tabela 2).

**Tabela 2:** Caracterização clínica e comportamental da amostra de pessoas com artrite reumatoide, Campo Grande, Mato Grosso do Sul, Brasil, 2024

Variáveis clínicas e comportamentais	n	%	IC95%
Tipo de AR (CID-10)			
Soro-positiva (M05)	54	56,3	46,9 – 66,7
Com comprometimento de outros órgãos e sistemas (M05.3)	1	1,0	0,0 – 3,1
Outras AR soro-positivas (M05.8)	16	16,7	9,4 – 25,0
Outras AR (M06)	14	14,6	8,3 – 21,9
Soro-negativa (M06.0)	2	2,1	0,0 – 5,2
Outras artrites reumatoides especificadas (M06.8)	2	2,1	0,0 – 5,2
Não especificada (M06.9)	7	7,3	2,1 – 12,5
Quantidade de comorbidades			
0	10	10,4	4,2 – 16,7
1	11	11,5	5,2 – 18,8
2	18	18,8	11,5 – 27,1
3	14	14,6	7,3 – 21,9
4	21	21,9	13,5 – 30,2
5	10	10,4	4,2 – 16,7
6	3	3,1	0,0 – 7,3
7	6	6,3	2,1 – 11,5
8	2	2,1	0,0 – 5,2
10	1	1,0	0,0 – 3,1
Dor com frequência			
Sim	69	71,9	61,5 – 80,2
Não	27	28,1	19,8 – 38,5
Atrapalha as atividades diárias			
Sim	57	59,4	50,0 – 68,8
Não	39	40,6	31,3 – 50,0
Tratamento não farmacológico			
Sim	41	42,7	33,3 – 52,1
Não	55	57,3	47,9 – 66,7
Psicólogo			
Sim	6	6,3	2,1 – 11,5
Não	90	93,8	88,5 – 97,9
Psiquiatra			
Sim	9	9,4	4,2 – 15,6
Não	87	90,6	84,4 – 95,8
Uso de cigarro			
Sim	17	17,7	10,4 – 25,0
Não	79	82,3	75,0 – 89,6
Uso de álcool			
Sim	12	12,5	6,3 – 19,8
Não	84	87,5	80,2 – 93,8

*n – frequência absoluta; % – frequência relativa percentual; IC95% – Intervalo de Confiança de 95%; CID-10 – Classificação Internacional de Doenças versão 10; AR – Artrite Reumatoide.*

Com a aplicação da Escala Visual Analógica de Dor, observou-se que a ausência de dor (0 pontos) foi a pontuação mais frequente (n = 24; 25,0%), seguida de 7 pontos (n = 14; 14,6%) e 8 pontos (n = 12; 12,5%). A pontuação média foi de 4,63 (desvio padrão = 3,41; mediana = 5,0; mínimo = 0,0; máximo = 10) (resultados não tabelados).

A maioria dos participantes não realizava tratamentos não farmacológicos nem possuía acompanhamento com psicólogos ou psiquiatras. No âmbito comportamental, a maioria não fazia uso de cigarro ou álcool.

Na Tabela 3 é possível observar a descrição de medidas de tendência central e de dispersão para os valores globais e por fatores de autocuidado e de complexidade da terapêutica farmacológica.

**Tabela 3:** Descrição das pontuações para o autocuidado e de complexidade da terapêutica farmacológica para a amostra de pessoas com artrite reumatoide, Campo Grande, Mato Grosso do Sul, Brasil, 2024

Variável	Média	Med.	DP	CV%	Mínimo	Máximo	P25	p75
ASAS-R Global	56,33	57,0	8,62	15,30	36	75	50,25	62,0
ASAS-R – Fator 1	24,31	24,0	4,44	18,26	8	30	22,0	28,0
ASAS-R – Fator 2	20,54	21,0	3,30	16,06	10	25	18,25	23,0
ASAS-R – Fator 3	11,48	11,0	3,57	31,09	4	20	9,0	14,0
ICFT Global	18,29	16,0	8,75	47,84	2	44	12,0	23,0
ICFT – Fator 1	3,05	3,0	2,28	74,75	1	11	1,0	5,0
ICFT – Fator 2	12,34	12,0	5,98	48,46	1	32	8,0	16,0
ICFT – Fator 3	2,96	2,0	2,98	100,6	0	12	1,0	4,0

*ASAS-R – Appraisal of Self-Care Agency Scale – Revised; ICFT – Índice de Complexidade da Farmacoterapia; Med – Mediana; DP – Desvio Padrão; CV – Coeficiente de variação percentual; p25 – Percentil 25 (1° quartil); p75 – Percentil 75 (3° quartil).*

Os resultados dos valores de p25 e p75 para adultos foram os seguintes: 11,0 e 22,0, para global; 1,0 e 5,0, para o Fator 1; 7,0 e 14,0 para o Fator 2; e 0,0 e 4,0 para o Fator 3. Para os idosos, foram identificados os valores a seguir: 13,0 e 25,5, para o valor Global; 1,0 e 5,0 para o Fator 1; 8,5 e 18,0 para o Fator 2; e 1,0 e 4,0 para o Fator 3. Com isso, em termos globais, verificou-se que a maior parte da amostra apresentou complexidade intermediária. O mesmo ocorreu quando se analisou separadamente as pontuações obtidas para o Fator 2 do ICFT. Para o Fator 1, a maior parte da amostra se enquadrou em baixa complexidade, ao passo que, para o Fator 3, a maior parte dos participantes esteve entre intermediária e alta complexidade (Tabela 4).

**Tabela 4:** Frequências dos níveis de complexidade farmacológica conforme ICFT Global e por fatores para a amostra de pessoas com artrite reumatoide, Campo Grande, Mato Grosso do Sul, Brasil, 2024

ICFT		Baixa Complexidade		Intermediária Complexidade			Alta Complexidade		
	n	%	IC95%	n	%	IC95%	n	%	IC95%
Global	29	30,2	21,9 – 39,6	46	47,9	37,5 – 58,3	21	21,9	13,5 – 31,3
Fator 1	46	47,9	38,5 – 58,3	22	22,9	14,6 – 31,3	28	29,2	19,8 – 37,5
Fator 2	26	27,1	18,8 – 36,5	44	45,8	35,4 – 55,2	26	27,1	17,7 – 35,4
Fator 3	30	31,3	21,9 – 41,6	33	34,4	25,0 – 43,8	33	34,4	25,0 – 43,8

*ICFT – Índice de Complexidade da Farmacoterapia; n – frequência absoluta; % – frequência relativa percentual; IC95% – Intervalo de Confiança de 95%.*

Conforme a Tabela 5, foram verificadas diferenças significativas nas pontuações medianas para o autocuidado nos Fatores 2 e 3 do ASAS-R. As diferenças pontualmente observadas se deram, respectivamente, entre os indivíduos com níveis Baixo e Alto no Fator 2 do ICFT (p = 0,042) e Intermediário e Alto para o ICFT Global (p = 0,011). Verificando-se valores de IC95% para as medianas em cada comparação em que se identificou diferença, observa-se que os intervalos se sobrepõem no caso dos resultados referentes ao Fator 2 do ASAS-R e Fator 2 do ICFT (Baixo = 18,00 – 21,00 *vs.* Alto = 20,51 – 24,00). Com isso, é possível que indivíduos com maior frequência de doses tenham maior poder para desenvolver seu autocuidado.

**Tabela 5:** Relação entre o autocuidado e complexidade da terapêutica farmacológica para a amostra de pessoas com artrite reumatoide, Campo Grande, Mato Grosso do Sul, Brasil, 2024

Variáveis do ICFT	Pontuação de autocuidado (ASAS-R) conforme Níveis de Complexidade Farmacológica						Valor de *p*
	Baixa		Intermediária		Alta		
	Média (DP)	Med.	Média (DP)	Med.	Média (DP)	Med.	
	ASAS-R Global						
Global	55,62 (7,88)	57,00	57,09 (9,19)	59,50	55,67 (8,56)	55,00	0,718*
Fator 1	55,78 (9,12)	57,50	55,91 (7,93)	57,00	57,57 (8,47)	58,00	0,669*
Fator 2	55,12 (7,65)	55,00	56,68 (8,83)	57,50	56,96 (9,36)	57,50	0,699*
Fator 3	57,73 (7,99)	59,50	56,64 (9,01)	58,00	54,76 (8,78)	54,00	0,384*
	ASAS-R – Fator 1						
Global	24,52 (3,81)	24,00	23,91 (5,13)	24,00	24,90 (3,67)	25,00	0,891**
Fator 1	23,67 (5,19)	24,00	25,05 (3,37)	25,00	24,79 (3,77)	25,50	0,713**
Fator 2	24,27 (3,96)	24,00	24,14 (4,47)	24,00	24,65 (4,97)	26,00	0,663**
Fator 3	25,47 (3,68)	26,50	24,33 (4,53)	25,00	23,24 (4,82)	24,00	0,167**
	ASAS-R – Fator 2						
Global	19,69 (3,54)	20,00	20,96 (2,78)	20,50	20,81 (3,92)	21,00	0,247**
Fator 1	20,26 (3,29)	20,00	20,41 (3,34)	21,00	21,11 (3,34)	22,00	0,462**
Fator 2	19,73 (2,76)	19,50	20,41 (3,49)	20,50	21,58 (3,32)	21,50	0,048**
Fator 3	20,17 (3,45)	20,00	20,82 (3,16)	21,00	20,61 (3,37)	21,00	0,731**
	ASAS-R – Fator 3						
Global	11,41 (2,59)	11,00	12,22 (3,88)	12,00	9,95 (3,68)	9,00	0,015**
Fator 1	11,85 (3,26)	12,00	10,45 (3,55)	10,00	11,68 (4,02)	11,00	0,307*
Fator 2	11,12 (3,05)	10,00	12,14 (3,72)	12,00	10,73 (3,73)	10,50	0,237*
Fator 3	12,10 (3,43)	12,00	11,48 (3,65)	11,00	10,91 (3,63)	10,00	0,422*

ICFT – Índice de Complexidade Farmacológica da Terapêutica; ASAS-R – Appraisal of Self-Care Agency Scale – Revised; *ANOVA um fator; **Kruskal-Wallis.

Por outro lado, sobre os resultados acerca da relação entre Fator 3 do ASAS-R e ICFT Global, esta sobreposição não ocorre (Intermediário = 11,00 – 14,00 *vs.* Alto = 8,00 – 10,00), o que reforça a existência da diferença nas pontuações entre os referidos grupos. Dessa forma, é possível que alta complexidade da farmacoterapia global favoreça o déficit de poder para o autocuidado.

## DISCUSSÃO

Algumas características sociodemográficas presentes na amostra deste estudo incluem faixa etária elevada, sexo feminino, nível de escolaridade fundamental, presença de comorbidades e dificuldades no controle da dor. Vale ressaltar que essas características já foram relatadas em outro estudo realizado em Portugal e podem estar relacionadas à complexidade da farmacoterapia, a qual pode impactar o autocuidado devido a prejuízos na autonomia funcional^(18)^.

A maioria dos indivíduos tinha 60 anos ou mais, estava em regime de polimedicamentos e apresentava múltiplas comorbidades. Evidências indicam que esses fatores parecem estar relacionados à ocorrência de problemas com a terapia medica-mentosa^(19)^, sendo que o uso de múltiplos medicamentos podem estar associado a comportamentos de não adesão^(20)^, além de levantar a possibilidade de também influenciar o autocuidado.

Vale destacar que a presença de comorbidades como hipertensão arterial e diabetes (incidência na amostra de 50% e 27%, respectivamente) pode aumentar a complexidade da farmacoterapia devido à introdução de diferentes formas farmacêuticas, frequências de dosagem e instruções adicionais^(21)^. Além da alta complexidade, outras dificuldades podem comprometer o tratamento farmaco-terapêutico e não são contempladas no ICFT, tais como: confusão entre os medicamentos, necessidade de lembretes e dependência de auxílio de um cuidador ou familiar responsável^(22)^.

A relação entre a pontuação do *ASAS-R Global* e do *ICFT Global* não foi estatisticamente significativa. Entretanto, foi identificada uma relação significativa entre o fator 2 (frequência das doses) do ICFT e o fator 2 (desenvolvendo poder para o autocuidado) do ASAS-R (*p* = 0,048). A diferença foi observada entre os níveis Baixo e Alto no fator 2 (frequência das doses) do ICFT e o fator 2 do ASAS-R (desenvolvendo poder para o autocuidado) (*p* = 0,042). Assim, sugere-se que uma alta frequência de doses tenha relação positiva com o autocuidado de indivíduos com AR.

Evidências indicam que pessoas com condições crônicas e outras comorbidades relatam dificuldades no dia a dia devido à necessidade de doses frequentes de medicação^(23)^. Apesar de ser percebido de forma negativa, um regime de tratamento com alta frequência de doses pode, na prática, favorecer o autocuidado.

Também foi observada uma relação significativa entre o *ICFT Global* e o fator 3 (faltando poder para o autocuidado) do *ASAS-R* (*p* = 0,015). Outra relação significativa foi identificada entre o fator 3 do *ASAS-R* (faltando poder para o autocuidado) e os níveis intermediário e alto do *ICFT Global* (*p* = 0,011). Sugere-se que um número elevado de medicamentos, maiores frequências de doses e um maior volume de informações adicionais possam contribuir para um estado de déficit de poder para o autocuidado.

A literatura aponta que a elevada complexidade do regime terapêutico pode dificultar a adoção de comportamentos de autocuidado^(24)^. Entretanto, entre idosos domiciliados, é possível que uma maior complexidade da terapia farmacológica seja benéfica no desenvolvimento do autocuidado e na adesão à medicação, devido ao apoio fornecido pela família na manutenção do tratamento medicamentoso^(25)^.

A alta complexidade da farmacoterapia foi associada a menor adesão medicamentosa^(26)^. A literatura destaca que uma maior adesão à medicação pode ser um fator de influência positiva no autocuidado^(27)^, sendo a adesão um elemento de potencial importância na análise dessa relação^(28)^.

Modificar os fatores da complexidade da farmacoterapia pode ser desafiador e deve contar com a interação da equipe multiprofissional, que desempenha um papel fundamental no processo de tomada de decisão sobre medicamentos^(29)^ O uso de estratégias de simplificação para a redução da carga de comprimidos e da frequência de dosagem tem sido relatado e apresenta dados positivos, indicando que a adesão e os resultados clínicos melhoram após a simplificação do regime, com destaque para os medicamentos injetáveis^(30)^. Além disso, a automatização do *ICFT* por meio de *software* pode otimizar sua utilização nas rotinas de atendimento multiprofissional, permitindo que qualquer profissional o utilize e obtenha resultados rapidamente para um direcionamento precoce da simplificação da terapia farmacológica em indivíduos com maior complexidade^(31)^.

Os valores encontrados por meio do *ASAS-R* representam um nível intermediário de autocuidado, semelhante ao identificado em outro estudo realizado com pacientes com fibromialgia (outra condição reumatológica)^(32)^. O provável obstáculo para que essa pontuação não tenha atingido um nível alto são os impactos da doença, que se manifestam principalmente sob a forma de dor, fadiga e deformidades articulares^(33)^. Esses fatores prejudicam a qualidade de vida, que, em indivíduos com AR, é inferior à da população geral^(34)^.

Para elevar o autocuidado, é necessário compreender que o estímulo está vinculado às habilidades de autogestão. Um indivíduo com crenças positivas em suas próprias capacidades pode gerenciar problemas específicos. A busca pela ausência de sintomas e o retorno às atividades diárias são fatores importantes que impulsionam o autocuidado^(35)^.

Pesquisas futuras em indivíduos com AR devem buscar desenvolver intervenções voltadas para a melhoria dos fatores de autocuidado avaliados pelo *ASAS-R* e da complexidade da farmacoterapia, além de avaliar a aplicação da estratégia de simplificação do regime terapêutico.

### Limitações do Estudo

Este estudo possui algumas limitações, sendo possível que os dados coletados não apresentem um alto nível de aleatoriedade. O aumento da amostra, em algum nível, pode ter reduzido essa limitação. Os dados não permitem determinar uma relação entre o ASAS-R Global e o ICFT Global; no entanto, a análise entre fatores identificou pontos de relação entre as escalas. Além disso, há um número limitado de estudos que busquem essa relação, o que impossibilitou a comparação com outros achados. Para algumas comparações, foi necessário recorrer a estudos sobre outras condições semelhantes à AR, como a fibromialgia.

### Contribuições para a área de enfermagem, saúde ou política pública

Na prática do cuidado em enfermagem, com foco no autocuidado, pessoas com alta frequência de doses apresentam maior fragilidade no desenvolvimento do autocuidado, e aquelas com níveis mais altos de complexidade farmacoterapêutica podem apresentar déficit de poder para o autocuidado.

Ao identificar indivíduos idosos, com múltiplas comorbidades, polifarmácia e alta complexidade farmacoterapêutica, o enfermeiro deve buscar alternativas para modificar esse nível de complexidade. Recorrer a intervenções com a equipe multidisciplinar e ao uso de estratégias de simplificação pode ser uma ferramenta útil para a prática da enfermagem.

No ensino, a implementação da análise da complexidade da farmacoterapia e do uso de intervenções para sua redução, tanto de forma teórica quanto prática, pode capacitar os acadêmicos para abordagens e resoluções de casos envolvendo alta complexidade farmacoterapêutica.

## CONCLUSÕES

Indivíduos com escores mais elevados de complexidade farmacoterapêutica (número de medicamentos, frequência de doses e informações adicionais) podem ter prejuízo no poder para o autocuidado. Por outro lado, uma maior frequência de doses, sem escores elevados para o número de medicamentos e informações adicionais, pode favorecer o desenvolvimento do autocuidado em indivíduos com AR.

A polifarmácia, as comorbidades e outras variáveis demográficas e clínicas podem estar negativamente relacionadas ao autocuidado e à complexidade farmacoterapêutica. Intervenções com a equipe multidisciplinar e o uso de estratégias de simplificação do regime terapêutico podem ser alternativas eficazes para reduzir o ICFT e melhorar o autocuidado de pacientes com AR.
